# Special Issue: “Machine Learning for Computer-Aided Diagnosis in Biomedical Imaging”

**DOI:** 10.3390/diagnostics12061331

**Published:** 2022-05-27

**Authors:** Seong K. Mun, Dow-Mu Koh

**Affiliations:** 1 Arlington Innovation Center: Health Research, Virginia Tech, Washington, DC 22101, USA; 2 Department of Radiology, The Royal Marsden Hospital, London SW3 6JJ, UK

The radiology imaging community has been developing computer-aided diagnosis (CAD) tools since the early 1990s before the imagination of artificial intelligence (AI) fueled many unbound healthcare expectations and other industries. Today we see rapid growth in the number of FDA-approved AI imaging products, and some products have been adopted in clinical settings. However, overall clinical adoption of most of the products has been relatively slow.

This special issue plans to focus on the challenges faced in evolving AI into more clinically meaningful for diagnostic imaging. The research in translational imaging AI requires a clear definition of clinical problems suitable to AI, improvement to the traditional convolution neural network (CNN), management of the image volume and quality in clinical settings, and the testing and integration of AI tools into clinical workflow. This special issue, Machine Learning for Computer-Aided Diagnosis in Biomedical Imaging, was organized with the paper on state-of-the-art concepts and practices in diagnostic imaging in radiology, pathology, and others, as summarized below. These papers address the common radiological or pathological areas of unmet needs, where a fully developed reliable AI tool would help to augment current practice or workflow. 

A review paper on automated segmentation of pelvic cancer discussed the critically important issues to bridge the gap between computer vision and patient care and pointed to the need for better input data quality, more common dataset for development, and testing of segmentation algorithms [1]. The paper on the three-dimensional MRI of the knee discusses a non-invasive method of segmenting relevant structures, such as cartilage, bone marrow lesions, and meniscus [2]. Additionally, the paper on applying deep learning in CT images for pulmonary nodule detection reviews multiple CNN architectures focusing on the segmentation and classification aspects for computer-aided lung cancer diagnosis [3]. Combining the advantages of CNN and transformers originating from the vision community can be used to address multi-modal image classification. This new method was applied to two data sets, parotid gland tumors and knee injury classifications, with improved performance [4]. Several papers deal with AI-based classifications, such as lung disease classification on chest X-ray images, colon tissue classification from whole slide images, brain hemorrhage classification in CT images, and white blood cell classification using multilevel CNN [5,6,7,8].

Two papers offer timely discussions on the use of AI for patient care. The use of AI to detect hospital-acquired thrombocytopenia in ICU post-operation [9]. In the other, AI was applied to CT images to screen and predict coronavirus infections with CT images [10].

A new three-step process for brain tumor classification consists of initial annotation of the region of interest, feature extraction using DenseNet-41, and classification using the novel CornerNet for Brain MRI analysis, with good performance when applied to two different datasets [11]. In addition to the studies described above, the use of AI in pathology is highlighted in the automatic assessment of glomerular pathology findings in Lupus Nephritis [12]. In addition, the social spider optimization-tuned neural network was applied to analyze microscopic images of breast tissue biopsy [13].

The use of deep learning with 3D optical coherence tomography images is an example of how these tools are becoming ubiquitous in all medical imaging. The paper evaluated four pre-trained CNN methods and concluded that EfficientNetB4 showed the best performance in identifying pathologic myopia from the 3D optical imaging system [14].

The total kidney volume is a biomarker to quantify disease progress of the autosomal dominant polycystic kidney disease. The biomarker analysis was carried out by integrating attention mechanisms, cosine loss function, and sharpness aware minimization to U-Net, which helped address the problems associated with the small number of MR images in the dataset [15].

In closing, while we noted significant and persistent progress in computer-aided diagnosis in diagnostic imaging, challenges do remain. We need more data sets with higher image quality. We need significant improvements in the science and technology of the convolutional neural network. We need to consider how we should integrate many AI tools into a clinical flow. There are significant barriers to the implementation of AI tools in real-world scenarios, not least the requirement for informatics support and development, which are often lacking in hospital systems. There also remains a need for a clear definition of what clinical problem AI should address. AI will eventually become an integral part of the digital transformation of diagnostic imaging that calls for the intimate and sustained collaboration of scientists, clinicians, engineers, and managers.
